# Non-Canonical Effects of ACTH: Insights Into Adrenal Insufficiency

**DOI:** 10.3389/fendo.2021.701263

**Published:** 2021-08-19

**Authors:** Valeria Hasenmajer, Ilaria Bonaventura, Marianna Minnetti, Valentina Sada, Emilia Sbardella, Andrea M. Isidori

**Affiliations:** Department of Experimental Medicine, Sapienza University of Rome - Policlinico Umberto I Hospital, Rome, Italy

**Keywords:** adrenal gland, adrenocorticotropic hormone, adrenal insufficiency, proopiomelanocortin, adrenal diseases, ACTH, POMC, extra-adrenal effects

## Abstract

**Introduction:**

Adrenocorticotropic hormone (ACTH) is produced from proopiomelanocortin, which is predominantly synthetized in the corticotroph and melanotroph cells of the anterior and intermediate lobes of the pituitary gland and the arcuate nucleus of the hypothalamus. Although ACTH clearly has an effect on adrenal homeostasis and maintenance of steroid hormone production, it also has extra-adrenal effects that require further elucidation.

**Methods:**

We comprehensively reviewed English language articles, regardless of whether they reported the presence or absence of adrenal and extra-adrenal ACTH effects.

**Results:**

In the present review, we provide an overview on the current knowledge on adrenal and extra-adrenal effects of ACTH. In the section on adrenal ACTH effects, we focused on corticosteroid rhythmicity and effects on steroidogenesis, mineralocorticoids and adrenal growth. In the section on extra-adrenal effects, we have analyzed the effects of ACTH on the osteoarticular and reproductive systems, adipocytes, immune system, brain and skin. Finally, we focused on adrenal insufficiency.

**Conclusions:**

The role of ACTH in maintaining the function of the hypothalamic–pituitary–adrenal axis is well known. Conversely, if we broaden our vision and analyze its role as a potential treatment strategy in other conditions, it will be evident in the literature that researchers seem to have abandoned this aspect in studies conducted several years ago. We believe it is worth re-evaluating the role of ACTH considering its noncanonical effects on the adrenal gland itself and on extra-adrenal organs and tissues; however, this would not have been possible without the recent advances in the pertinent technologies.

## Introduction

Adrenocorticotropic hormone (ACTH) was first described in 1933, and after nearly 20 years, it was demonstrated that this polypeptide hormone stimulates adrenocortical activity (1–3). ACTH is exclusively produced from prohormone proopiomelanocortin (POMC), which is majorly synthetized in the corticotroph and melanotroph cells of the anterior and intermediate lobes of the pituitary gland and the arcuate nucleus of the hypothalamus. ACTH synthesis has also been described in other organs, such as the skin (4). After synthesis and folding, POMC is transported in vesicles and processed in secretory granules before ultimately reaching the plasma membrane (5). The selective cleavage of POMC by prohormone convertase (PC) and the timing of secretion are cell-specific and follow the regulated secretory pathway, along with other hormones (6) because of a highly conserved sorting signal motif (7). In immature secretory granules, PC1/3 and PC2 enzymes process POMC. In the anterior pituitary lobe, PC1/3 is responsible for the posttranslational cleavage that generates 16-kDa N-POMC, ACTH, and β-lipotropin. In the intermediate pituitary lobe and hypothalamus, a more complex processing of POMC, including PC2 activity along with other enzymes, generates more active peptides, such as α-melanocyte-stimulating hormone and β-endorphin (8).

After its cleavage from POMC by PC1/3, ACTH is secreted by mature granules from the anterior lobe of the pituitary gland into the circulation, targeting its receptor on peripheral cells, the melanocortin 2 receptor (MC2R). In addition to MC2R, which is highly specific for ACTH, other melanocortin receptors (MCRs; MCR1, MCR3, MCR4, and MCR5) can bind to ACTH and other POMC-derived peptides. These receptors are expressed in various tissues (**Table 1**). MC2R is part of the melanocortin receptor family, a group of class A G-protein-coupled receptors (GPCRs) that share binding to melanocyte-stimulating hormone peptides (9) and are the smallest GPCRs known. In contrast to other MCRs, MC2R binding requires melanocortin-2 receptor accessory protein 1 (MRAP) for proper migration to plasma membrane and receptor-ligand complex formation and downstream signaling (10). The function of MRAP is crucial to ACTH function, and mutations in its gene cause ACTH resistance syndrome and type 2 familial glucocorticoid deficiency (10).

**Table 1:** Melanocortin receptors (MCRs) and main sites of expression.

Receptor	Tissues
MC1R	Melanocytes and immune cells
MC2R	Adrenal cortex, adipocytes, testis, prostate, endometrium, and immune cells
MC3R	Hypothalamus, limbic system, immune cells, placenta, gut, and kidney
MC4R	Hypothalamus, limbic system, brain, spinal cord, and immune cells
MC5R	Muscles, liver, spleen, lungs, brain, and adipocytes

Similar to other GPCRs, MC2R activation leads to an increase in intracellular cyclic adenosine monophosphate (cAMP) stimulating the protein kinase A (PKA) signaling pathway (11). Other intracellular pathways activated by ACTH are mitogen-activated protein kinase (12) and cAMP response element-binding protein (13). A role of calcium influx that cooperates with ACTH-mediated steroid synthesis has also been identified, and many other secondary messengers are involved in ACTH downstream signaling, although their independence from the PKA pathway is still under discussion (14). The interaction between MC2R and ACTH in the adrenal glands leads to transcription of genes responsible for steroidogenesis, such as the steroidogenic acute regulatory protein (StAR) (14).

**Figure 1:**
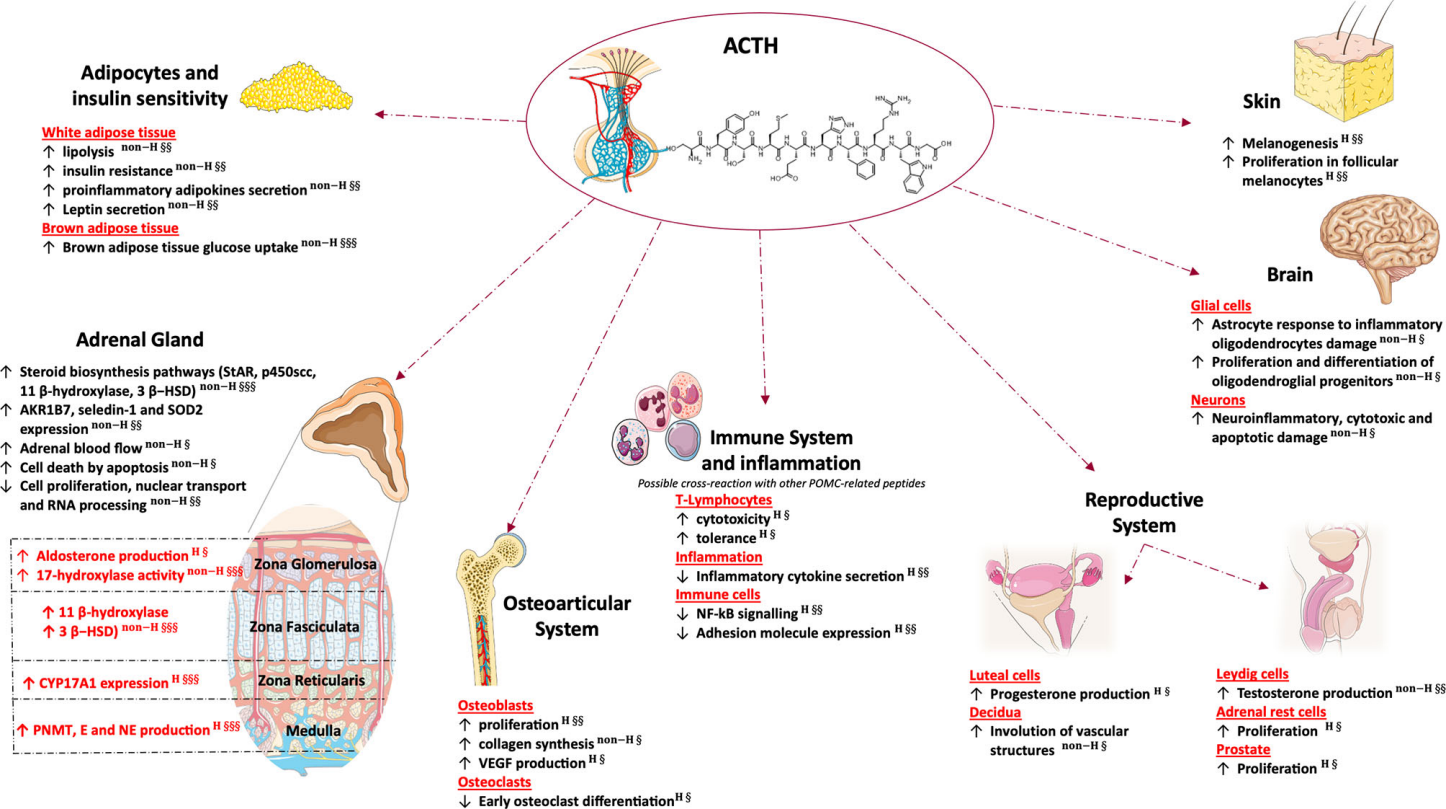
Glucocorticoid independent effects of adrenal and extra-adrenal ACTH. This figure was created using Servier Medical Art templates, which are licensed under a Creative Commons Attribution 3.0 Unported License; (https://smart.servier.com). 3β- HSD, 3β-Hydroxysteroid dehydrogenase; AKR1B7, Aldo-keto reductase family 1 member B7; E, epinephrine; NE, norepinephrine; NF-kB, nuclear factor kappa-light-chain-enhancer of activated B cells; P450scc, cholesterol side-chain cleavage enzyme; PNMT, phenylethanolamine N-methyltransferase; Seledin-1, selective Alzheimer disease indicator 1; SOD2, superoxidase dismutase 2; StAR, steroidogenic acute regulatory protein; VEGF, vascular endothelial growth factor. H, human study; non-H, non-human study; §, Published post-2010; §§, Published between 2000 and 2009; §§§, Published prior to 2000.

Aim of this review is to provide an overview of the effects of ACTH, focusing on the impact of extra-adrenal and non-canonical signaling in health and disease. First, we will briefly summarize evidence on the impact of ACTH signaling on glucocorticoid, mineralocorticoid and androgen secretion, the role in mediating adrenal growth and development and the effects on steroidogenesis and gene expression. Then, we will describe the effects of ACTH on the osteoarticular and reproductive systems, adipocytes, immune system, brain and skin (**Figure 1**). Lastly, we will provide an overview of the role of ACTH in adrenal disorders, focusing on adrenal insufficiency and suggesting possible implications of its excess and defect.

We performed a research of published literature with no time constraints. Only English papers were included. When possible, results from human studies were preferred over *in vitro* or animal studies. Being out of the main scope of this review, papers focusing on ACTH as a diagnostic marker for adrenal diseases were excluded.

## Adrenal Effects of ACTH

### Corticosteroid Rhythmicity

It is well established that circadian changes in ACTH and glucocorticoids are associated with expression of clock-related genes (15–19). In adrenal tumors, the clock machinery that mitigates the response to ACTH and stress favors a higher and more arrhythmic corticosteroid secretion when dysregulated, suggesting that hypercortisolism exerts effects on circadian genes, contributing to the worsening of disease-related comorbidities (16, 20, 21). Circadian gene expression is dysregulated in patients with adrenal insufficiency and normalize under more physiological timing of glucocorticoids, emphasizing the importance of synchronization of clocks to coordinate the endogenous and exogenous signals to achieve cellular homeostasis (22, 23).

### Effects on Steroidogenesis

ACTH is the key hormone controlling steroidogenesis in the adrenal gland, inducing responses in both the short and long terms. Acute and chronic ACTH stimulation lead to the mobilization of cholesterol, the first substrate for steroidogenic pathway (24), owing to the increase in StAR gene transcription (25, 26). StAR catalyzes the conversion of cholesterol to pregnenolone, the first and limiting step of steroidogenesis. Other steroidogenic enzymes, such as P450scc, the mitochondrial cholesterol side-chain cleavage enzyme that catalyzes conversion of cholesterol to pregnenolone, and P450C11 (11beta-hydroxylase), which catalyzes the transformation of deoxycorticosterone to corticosterone and that of 11-deoxycortisol to cortisol (25), are responsive to chronic ACTH stimulation. Through CYP17A1, ACTH promotes the secretion of androgen precursors, dehydroepiandrosterone (DHEA), its sulphated derivative DHEAS and androstenedione (27). Furthermore, an increase in size of the adrenal gland occurs after chronic ACTH administration, suggesting that this hypertrophy is a consequence of steroidogenesis enhancement (28). Under physiological conditions, ACTH mainly controls cortisol and androgen precursors secretion, even though the concept that aldosterone production is independent of ACTH is now outdated. As ACTH binding to MC2R also stimulates aldosterone secretion in addition to cortisol, even doses of ACTH within the physiological range can induce aldosterone synthesis (29–32) through a slow, but sustained, Ca^2+^ influx (33). In fact, ACTH binding to MC2R results in the production of second messengers (cAMP together with Ca^2+^ influx). This pathway, through positive feedback loops, enhances steroid secretion (14). Different exposures to ACTH might affect aldosterone production differently; in fact, under continuous intravenous ACTH infusion aldosterone increases and then returns to basal levels within 72 hours, but pulsatile administration of ACTH, which mimics its physiological release, causes aldosterone to remain at high levels (34). However, most results on strong aldosterone stimulation by ACTH are limited to *in vitro* studies (14) while evidence from animal models suggest an inhibitory effect of Angiotensin II signaling on cAMP and Ca^2+^ intracellular cascades, that are activated by ACTH binding to MC2R, thus dampening the *in vivo* effects on mineralocorticoid secretion.

### Effects Against Reactive Oxygen Species

ACTH-induced steroidogenesis reactions involve lipid peroxidation and production of reactive aldehyde metabolites that generate reactive oxygen species (ROS) and thus a considerable cellular oxidative stress. Consequently, several enzymes involved in the detoxification are mobilized in adrenal cells. Aldo–keto reductases participate into this detoxification process, and their expression is ACTH-dependent (35). Moreover, in human and rat adrenal cells, seladin-1 (selective Alzheimer disease indicator 1, also named 24-dehydrocholesterol reductase) expression and redistribution to the nucleus occur after ACTH treatment (36). Finally, increased expression of SOD2, (mitochondrial superoxidase dismutase 2, a metalloenzyme involved in the scavenging of mitochondrial ROS) is also induced by ACTH (37). Therefore, ACTH regulates the expression of enzymes responsible for steroid biosynthesis and nonsteroidogenic enzymes involved in preventing ROS-induced cell toxicity.

### Effects on Adrenal Growth and Adrenal Blood Flow

An *in vitro* study showed that ACTH leads to increased cell death through the apoptosis of isolated cells in cultures of the adrenal cortex; however, the zona fasciculata and zona reticularis are more resistant to the cytotoxic and antimitogenic effects of ACTH than zona glomerulosa (38). In contrast with these results, in animal models, ACTH regulates adrenal gland trophicity (28, 39) and increases adrenal blood flow (40). Furthermore, glucocorticoid-induced suppression of ACTH inhibits cell proliferation, induces apoptosis, decreases adrenal weight and cellularity of the adrenal cortex, and triggers vascular changes through loss of vascular endothelial growth factor protein expression, thereby causing regression of the vascular network (39). Moreover, knockout of the MC2R gene in mice leads to marked atrophy of the zona fasciculata (41) and high levels of MC2R expression in the undifferentiated zone, which contains stem cells supports the notion that ACTH may play an important role in adrenal cell differentiation and the importance of the ACTH–MC2R complex in adrenal development (42). Differences between *in vitro* cell cultures and *in vivo* models with regard to cell death by apoptosis are attributable to intraglandular factors, such as autonomic innervation and vascularization, supporting the importance of structural integrity and compartmentalization of the adrenal gland and suggesting that ACTH is primarily a differentiation factor that controls steroid secretion rather than a proliferative factor (38, 43, 44).

### Effects on Gene Expression

Different expression of key steroidogenic enzymes lead to zone-specific production of steroid hormones responsible for a distinct steroidogenic phenotype among the three constituent zones of the adrenal cortex (45–47). The Y1 mouse adrenal-cortical cell line, a model of normal mouse adrenal cortex cells, has been used to determine the effects of ACTH on gene expression. After ACTH stimulation, the levels of various transcripts were measured after 24 h (48). ACTH increased gene expression involved in cholesterol synthesis and mobilization and steroidogenic enzyme synthesis, confirming the importance of this hormone in the steroidogenic biosynthesis pathway. Conversely, >50% of studied transcripts were downregulated and affected DNA replication, mitotic cell cycle, and RNA processing and nuclear transport, suggesting a possible modulatory effect of ACTH on adrenal cortex cell growth (48).

### Effects on Adrenal Medulla

Although ACTH is known to be most effective on the adrenal cortex, some studies suggest that it can also influence the adrenal medulla. Hypophysectomy induces a decrease in both the adrenal epinephrine content and phenylethanolamine N-methyltransferase (PNMT)—which converts norepinephrine to epinephrine—in rats. These alterations were restored upon ACTH injection (49, 50). The increase in PNMT by long-term ACTH treatment also occurs in healthy rats, suggesting that ACTH-induced adrenal stimulation influences the enzyme activities of adrenal medulla (51). Moreover, after ACTH stimulation test, both epinephrine and norepinephrine increased in adrenal venous blood in humans through ACTH-induced increase in blood flow and enzyme activities (52). These mechanisms are possibly mediated by ACTH-induced increase in adrenal medulla exposure to glucocorticoids, that are active inductors of PNMT (53).

## Extra-adrenal effects of ACTH

### Osteoarticular System

Conventionally, ACTH activity through the increase in glucocorticoid level has a detrimental impact on bone mass, resulting in bone loss and osteoporosis. This is only true in endogenous or exogenous glucocorticoid excess (54, 55); at physiological levels, cortisol does not appear to have a negative effect on bone differentiation or proliferation (56). The prevalence of osteoporosis seems higher in patients with adrenal Cushing’s syndrome, presenting typically with ACTH suppression, than in those with Cushing’s disease (55, 57–59). These effects are undoubtedly due to adrenal androgen reduction derived from low ACTH levels, but a protective role of ACTH on bone has also been proposed. Osteoblasts express high-affinity ACTH receptors. Furthermore, high-dose ACTH stimulates osteoblastic proliferation and significantly increases osteoblast collagen synthesis (60–62). Conversely, lower ACTH doses seem to oppose osteoblast differentiation (60). ACTH stimulates VEGF production from osteoblasts and inhibits osteoclast differentiation *in vitro* (63, 64). Notably, murine osteoclasts are able to synthesize and release ACTH (61), so locally produced ACTH might also regulate bone metabolism. Finally, ACTH administration was proposed as a therapy for osteoarticular inflammatory conditions (65), for example it is included in guidelines for gout management (66), not only because of ACTH-induced glucocorticoid production but also because a direct role of ACTH was speculated (67, 68).

### Reproductive System

*Males*. MC2R receptor is expressed in mouse fetal testis, in both gonocytes (69) and Leydig cells (70, 71). The testis MC2Rs seem to be functionally active and stimulate testosterone production in fetal and neonatal mice in a dose-dependent manner; however, the effect is lost in postpubertal mice (70), suggesting that ACTH regulates testosterone production in fetal but not in adult testes. The importance of ACTH signaling in the development of testicular adrenal rest tumors (TART) in conditions characterized by early exposure to increased levels of adrenocorticotropin will be discussed in the section on adrenal disorders. Data on the prostate are scarce. Prostate activated MC2R seems to promote cell proliferation in a concentration-dependent manner (72). Therefore, targeting MC2R signaling has been proposed as a novel strategy for prostate carcinoma treatment (73). Interestingly, local administration of ACTH1–24 into the hypothalamic periventricular region of male rats induced penile erection *via* melanocortin-3 receptor (74).

*Females*. During a clinical trial on female premenopausal patients with Addison’s disease, menstrual disturbances were recorded in four of nine patients after administration of high-dose tetracosactide (ACTH1-24) (75). Robust MC2R expression was found in the glandular epithelium and lesser degree in stromal cells of human endometrium, suggesting the direct role of ACTH in regulating endometrial glandular secretion (76). Moreover, high ACTH1-24 concentrations promote involution of vascular structures in cultured decidua (76). MC2R is also expressed in bovine and rabbit ovaries, and a direct effect of ACTH on ovarian steroidogenesis was proposed (77, 78). However, despite *in vitro* studies have shown promising results in animal models, human studies are lacking. Most clinical trials on the role of ACTH in female reproduction have focused on its effects on androgen production in polycystic ovary syndrome (PCOS), and results suggested a possible contribution of dysregulated adrenal steroidogenesis in the pathogenesis of hyperandrogenism in PCOS (79).

### Adipocytes and Insulin Sensitivity

The melanocortin system plays a crucial role in energy expenditure (80), but the direct role of ACTH is still unclear. MCR2 mRNA is expressed in murine adipocytes (81, 82). ACTH can stimulate lipolysis *in vitro* in rodent adipose tissue through MC2R-dependent cAMP/PKA activation (80, 83). MRAP seems to play a critical role in the regulation of ACTH-induced lipolysis (84), and insulin resistance and glucose intolerance have been shown in MRAP2 knockout mice (85). However, ACTH does not seem to have a role in human adipose lipolysis (86, 87).

ACTH also seems to improve brown adipose tissue function in obese rat and mouse, an effect opposed by corticosterone. Moreover, ACTH increases glucose uptake in isolated brown adipocytes in the absence of insulin (88–90). Conversely, in cultured white mouse adipocytes, ACTH has been shown to directly induce insulin resistance and increase pro-inflammatory adipokine expression (82). Moreover, it has been found that ACTH is also a potent inhibitor of leptin expression (91). Waist circumference, prevalence of diabetes mellitus, and dyslipidemia are not significantly different in patients with pituitary or adrenal Cushing’s syndrome (55, 92). To our knowledge, no clinical study has evaluated direct metabolic effects of ACTH in humans, either in physiological or pathological conditions.

### Immune System and Inflammation

When analyzing the effects of ACTH on immunity, it is imperative to identify the distinction between glucocorticoid-mediated and glucocorticoid-independent effects. In fact, glucocorticoids are powerful modulators of the immune system (93), but some effects of ACTH and other POMC-related peptides are independent from the adrenal response to corticotropin. MCRs have been described in most immune lineages (94), including T and B lymphocytes, CD14^+^ monocytes, natural killer cells, and granulocytes. The activation of MCRs, and particularly of the MC3R, has anti-inflammatory effects, including modulation of T lymphocyte cytotoxicity and tolerance (95, 96) and inhibition of NF-κB signaling, which appears to mediate a reduction in inflammatory cytokine secretion and adhesion molecule expression (97–99). However, data on the direct impact of ACTH on inflammation are lacking, mostly due to the promiscuity of other MCRs. In fact, although MC2R is selective for ACTH, the possibility of cross-binding of other MCRs, such as MC3R (100), on immune cells has decreased the reliability of studies on MC2R^−/−^ mice. Very recently, MC2R expression was confirmed on T regulatory Lymphocytes, and ACTH therapy was shown to promote allograft acceptance after heart transplant in mice (101). This supports a tolerogenic role of ACTH in inflammation, that would partially explain the efficacy in inflammatory diseases such as gout.

### Brain

MC4R activation displays neuroprotective and neuroregenerative effects in several models of animal neurodegenerative diseases (102). ACTH has been found to promote oligodendrocyte protection (103) and neuron protection from inflammatory, excitotoxic, and apoptotic damages (104, 105). Dated research showed that rat embryo neurons respond to ACTH displaying a denser neuritic network (106) and displayed neuroprotective effects after hemorrhage insult by activating anti-inflammatory pathways (107, 108). Melanocortins have been proposed as therapy for brain injuries and several chronic neurodegenerative disorders (102). In epilepsy, ACTH is effective against infantile spasms, and it is administered to children suffering from intractable seizures and West syndrome (109, 110). Although the specific mechanisms for this antiepileptic effect remain unknown, beyond the **“**conventional**”** glucocorticoid production, ACTH seems to have a direct modulation of amygdala neurons leading to decreased production of the proconvulsant peptide CRH (110). Also, few data show that patients relapsing forms of multiple sclerosis may benefit from ACTH gel (111).

### Skin

MC1R, expressed in melanocytes, plays a crucial role in the regulation of cutaneous pigmentation binding α-melanocyte-stimulating hormone (α–MSH) and ACTH with the same affinity, with a dose dependent stimulation of melanogenesis and melanocytes proliferation (112–114). MCR1 extensive polymorphism is considered the major contributor to the diversity of human pigmentation (115). Interestingly, ultraviolet (UV) radiation exposure activates transcription of both keratinocytes and melanocytes POMC gene, resulting in increased local production of α -MSH and ACTH, suggesting that they act as paracrine and autocrine regulators to protect the skin from UV damage (116, 117).

## Adrenal Disorders

Disorders of glucocorticoid secretion involve downstream or upstream dysregulation of ACTH secretion or activity. Even though several differences have been described between ACTH-dependent and independent hypercortisolism, chronic adrenal insufficiency (AI) is characterized by prolonged exposure to ACTH excess or defect without increased endogenous glucocorticoids or androgens (118), providing a valuable model for speculating on the effects of ACTH. Therefore, it will be the focus of this section.

Primary adrenal insufficiency (PAI) is characterized by adrenal failure, leading to insufficient cortisol secretion and increased ACTH levels (118). The increased ACTH and related peptide levels lead to skin hyperpigmentation, one of the pathognomonic characteristics of PAI, due to hyperstimulation of melanocytes. Aside from the effects on the skin, little is known on the systemic effects of increased ACTH in these patients. Conversely, patients with secondary adrenal insufficiency (SAI) have low or inappropriately normal ACTH levels, often due to pathologic processes or interventions involving pituitary or sellar or parasellar regions and leading to multiple hormone deficiencies (118). Another common cause of adrenal insufficiency (AI) is prolonged exposure to exogenous glucocorticoids, which is characterized by isolated low ACTH levels due to the suppression of physiological hypothalamic–pituitary–adrenal (HPA) function.

### Genetic Forms of Adrenal Insufficiency

Most genetic causes of HPA axis impairment are rare, and data are obtained by case reports or case series. Defects in ACTH synthesis, receptor, or signaling are among the mechanisms underlying genetic forms of AI. As previously mentioned, resistance to ACTH binding at the adrenal glands can be caused by mutations of the MC2R or MRAP gene and lead to type 1 and type 2 familial glucocorticoid deficiency, respectively. Alterations in ACTH synthesis include syndromes associated with pituitary hypoplasia or aplasia and isolated ACTH deficiency due to the disruption of POMC, PC1, or TPIT, the transcription factor responsible of POMC synthesis in the corticotroph pituitary cells (118).

Generally, the presenting signs and symptoms are hypoglycemia and jaundice due to AI, usually developing during the neonatal period or infancy although the age of onset is variable (119). Failure to thrive, seizures, and frequent infections are also common features. Patients with defective ACTH receptor or signaling present with hyperpigmentation and an unusually tall stature (120) due to the accumulation of ACTH and other POMC metabolites. Other similar genetic causes of AI characterized by increased ACTH and other POMC metabolites include defective steroidogenesis, peroxisome defects, mitochondrial defects, adrenal dysgenesis, and impaired redox homeostasis (118).

In contrast, in diseases affecting POMC synthesis ACTH levels are low or undetectable, and although these syndromes share AI with those formerly described, they differ under many other aspects. Interestingly, TPIT disruption only affects POMC production from the pituitary gland, whereas other sites of synthesis, such as the skin and hypothalamus, seem preserved (121); affected patients lack other signs and symptoms that are present in POMC defects. In fact, patients with monogenic POMC and PC1 defects show altered pigmentation and auburn hair, due to the lack of α-melanocyte-stimulating hormone, and hyperphagic obesity usually presenting during infancy, due to defective POMC hypothalamic signaling (119).

### Acquired Adrenal Insufficiency

The acquired forms of PAI and SAI are more common and better studied than their genetic counterparts. However, data on the contribution of ACTH in establishing the clinical picture and prognosis, aside from adrenal effects and canonical hyper- or hypo-pigmentation, are still limited. In male patients affected by congenital adrenal hyperplasia, prolonged and early exposure to high ACTH levels can lead to the development of testicular adrenal rest tumors (122), probably due to the proliferation of totipotent steroidogenic cells, which can ultimately cause infertility and altered testicular hormone secretion (122). It has been suggested that early exposure to high ACTH levels is crucial for the proliferation of pluripotent cells into TART (122). In some cases, a similar clinical picture has been described in other forms of PAI such as adrenoleukodystrophy (123).

## Discussion

The pleiotropic effects of ACTH are well known. However, its use as a therapeutic agent in the clinical practice is currently limited to selected diseases such as infantile spasms or anti-inflammatory resistant gout, as previously described. In these conditions, especially in infantile spasms in which ACTH treatment is more broadly used, therapeutic effects and treatment-related adverse events largely rely on increased glucocorticoid secretion secondary to ACTH stimulation of the adrenal cortex (124). Furthermore, no conclusive evidence is available on the effects of its excess and defect in AI.

Even under optimal glucocorticoid replacement, patients affected by PAI show decreased quality of life (125) and increased mortality, with infections and cardiovascular and respiratory diseases as leading causes of death (126–128). In patients with hypopituitarism, AI has been associated with increased risk of death (129), and among patients with AI, those with SAI showed higher mortality (130); however, this result has not been confirmed by a more recent study (127). Cardiovascular mortality in patients with AI has been reported to be higher in female patients (128) and is associated with former comorbidities (131). Moreover, despite the presence of normal visceral fat, patients with PAI have an increased number of cardiovascular risk biomarkers (132). As previously described, *in vitro* studies have yielded confounding results on the effects of ACTH on insulin sensitivity and adipose tissue. Given the lack of conclusive data, studies on the possible role for ACTH excess in metabolic complications of PAI could provide useful insights.

Recent studies have shown immune alterations in patients with PAI and SAI (23, 133), which were partially restored by replacement therapies more respectful of circadian cortisol profile (22, 23). Even though subgroup analyses did not show any differences in outcomes after switch to modified release hydrocortisone between PAI and SAI (23), the study from Isidori and colleagues was not powered to investigate specific effects of ACTH on immune function in AI at baseline or after therapy switch, therefore possible differences could have been unrecognized. More studies are necessary to investigate this aspect.

## Conclusions

The role of ACTH in maintaining adrenal homeostasis and participating in the HPA axis is self-evident. However, after an initial number of studies on its potential as a therapeutic strategy in many diseases and conditions, researchers seem to have abandoned the “corticotropin path” and have focused more on its downstream hormone pathways (glucocorticoids and androgens). Owing to the advances in knowledge and methodologies, it is time to rediscuss the role of ACTH in affecting general outcomes in adrenal diseases and the possible use of its noncanonical effects to address unmet needs.

## Author Contributions

VH: substantial contributions to conception of the manuscript, analysis and interpretation of data, drafting the article, revising it critically, and final approval of the version to be submitted. IB, MM, ES, and VS: substantial contributions to conception of the manuscript, analysis and interpretation of data and drafting the article. ES: substantial contributions to revising the manuscript critically for important intellectual content, and final approval of the version to be submitted. AI: substantial contributions to conception and design of the manuscript, drafting the article and revising it critically for important intellectual content, and final approval of the version to be submitted. All authors contributed to the article and approved the submitted version.

## Funding

The project was partially funded by the CHRONOIMAGE project (PRIN 2017HRTZYA) by MIUR.

## Conflict of Interest

AI has served as a consultant in the advisory boards for Novartis, Takeda, Recordati, and Sandoz and has received unconditional research grants from Shire, IPSEN, and Pfizer.

The remaining authors declare that the research was conducted in the absence of any commercial or financial relationships that could be construed as a potential conflict of interest.

## Publisher’s Note

All claims expressed in this article are solely those of the authors and do not necessarily represent those of their affiliated organizations, or those of the publisher, the editors and the reviewers. Any product that may be evaluated in this article, or claim that may be made by its manufacturer, is not guaranteed or endorsed by the publisher.
